# Key Role of Microglial Matrix Metalloproteinases in Choroidal Neovascularization

**DOI:** 10.3389/fncel.2021.638098

**Published:** 2021-02-26

**Authors:** Juhee Kim, Jong-Heon Kim, Ji Yeon Do, Jung Yi Lee, Ryoji Yanai, In-kyu Lee, Kyoungho Suk, Dong Ho Park

**Affiliations:** ^1^Leading-edge Research Center for Drug Discovery and Development for Diabetes and Metabolic Disease, Kyungpook National University Hospital, Daegu, South Korea; ^2^Brain Science and Engineering Institute, Kyungpook National University, Daegu, South Korea; ^3^R&D Center, JD Bioscience Inc., Gwangju, South Korea; ^4^Department of Ophthalmology, Yamaguchi University Graduate School of Medicine, Ube, Japan; ^5^Department of Internal Medicine, School of Medicine, Kyungpook National University, Kyungpook National University Hospital, Daegu, South Korea; ^6^Research Institute of Aging and Metabolism, Kyungpook National University, Daegu, South Korea; ^7^Department of Pharmacology, School of Medicine, Kyungpook National University, Daegu, South Korea; ^8^Department of Ophthalmology, School of Medicine, Kyungpook National University, Kyungpook National University Hospital, Daegu, South Korea

**Keywords:** age-related macular degeneration (AMD), choroidal neovascluarization, matrix metalloproteinase, microglia, aqueous humor

## Abstract

Age-related macular degeneration (AMD), especially neovascular AMD with choroidal neovascularization (CNV), is the leading cause of blindness in the elderly. Although matrix metalloproteinases (MMPs) are involved in pathological ocular angiogenesis, including CNV, the cellular origin of MMPs in AMD remains unknown. The present study investigated the role of microglial MMPs in CNV. MMP activities were analyzed by gelatin zymography in aqueous humor samples from patients with CNV and laser-induced CNV mice. Active MMP-9 was increased in the aqueous humor samples from neovascular AMD patients compared with control subjects. In the retinal pigment epithelium (RPE)/choroid from CNV mice, active MMP-9 increased, beginning 1 h post-CNV induction, and remained upregulated until Day 7. In RPE/choroid from CNV mice, active MMP-9 was suppressed by minocycline, a known microglial inhibitor, at 6 h and 1-day post-CNV induction. Flow cytometry revealed that the proportion of activated microglia increased very early, beginning at 1 h post-CNV induction, and was maintained until Day 7. Similarly, immunohistochemistry revealed increased microglial activation and MMP-9 expression on CNV lesions at 6 h and 1-day post-CNV induction. SB-3CT, an MMP inhibitor, decreased vascular leakage and lesion size in laser-induced CNV mice. These findings indicated nearly immediate recruitment of activated microglia and very early MMP-9 activation in the RPE/choroid. The present study newly identified a potential role for early microglial MMP-9 expression in CNV, and furthermore that modulating microglial MMP expression is a novel putative therapeutic for CNV.

## Introduction

Age-related macular degeneration (AMD) is the leading cause of blindness in the elderly (Gehrs et al., 2006). Its prevalence is expected to increase as aging populations expand, with approximately 288 million people worldwide affected by AMD by 2040 (Wong et al., 2014). Neovascular AMD is characterized by the invasion of neovessels originating from the choroid through breaks in the Bruch’s membrane into the subretinal space in the process of choroidal neovascularization (CNV). CNV occurs during late-stage advanced AMD and can lead to severe vision loss (Ferris et al., 2013).

For pathological angiogenesis to occur, the basement membrane surrounding endothelial tubes must be degraded to facilitate migration and proliferation of endothelial cells (Hanahan and Folkman, 1996), which is modulated in part by matrix metalloproteinases (MMPs; Pepper, 2001). MMPs are extracellular endopeptidases that cleave extracellular matrix proteins to regulate pathological tissue remodeling in disease states, including inflammation (Nagase and Woessner, 1999). Prior studies reported that in normal tissues, basal MMPs are absent or weakly expressed (Pepper, 2001). MMPs are upregulated in endothelial cells and immune cells in response to a variety of pathological events, including inflammation and angiogenesis (Lambert et al., 2002; O’Grady et al., 2007). Among the large MMP family, MMP-2 and MMP-9 are of particular interest to CNV, as their substrates include type IV collagen, a component of the Bruch’s membrane (Alcazar et al., 2007). In the murine laser-induced CNV model, MMP-9 and MMP-2 expression are upregulated on Days 3 and 5 post-CNV induction, when neovascular lesions are already present, and synergistically promote CNV in this context (Lambert et al., 2002, 2003). However, potential early changes of MMP expression prior to 3 days post-CNV, and therefore the role of MMPs in initiation of the angiogenic response, have not been reported. Further, the cell types that secrete active MMP-9 during early CNV formation have not been identified, which is paramount to the development of targeted approaches to treatment.

Microglia are resident immune cells in the retina that significantly contribute to the inflammatory processes of age-related retinal pathologies and are known sources of MMPs (Chen and Xu, 2015). In the healthy retina, inactive microglia retain a stable state characterized by a ramified morphology. However, in response to injury or degeneration, microglia are activated, assuming an amoeboid morphology and migrating to disease lesions (Karlstetter et al., 2015). This has been observed in pathological retinal angiogenesis (Connor et al., 2007), retinal detachment (Okunuki et al., 2018), retinal degeneration (Peng et al., 2014), and autoimmune uveitis (Okunuki et al., 2019). Because activated microglia affect lesion sites by creating an inflammatory microenvironment, modulating microglial function is a potential therapeutic approach for inflammatory chorioretinal diseases (Aslanidis et al., 2015).

Although previous studies have reported the effect of tetracycline derivatives, including doxycycline, in CNV, these studies focused on FasL expression in the retinal pigment epithelium (RPE; Roychoudhury et al., 2010) or macrophage infiltration (Huang et al., 2013), rather than MMPs. Moreover, although minocycline is a known microglial inhibitor (Yrjanheikki et al., 1999), the mechanism behind this inhibition has not been fully elucidated, and the effect of minocycline on the microglial MMP secretion has not yet been evaluated. We, therefore, hypothesized in the present study that CNV disease severity could be alleviated by suppressing microglial MMP activity.

## Materials and Methods

### Experimental Animals

All animal experiments were conducted following the guidelines of the Association for Research in Vision and Ophthalmology Statement for the Use of Animals in Ophthalmic and Vision Research and were approved by the Animal Care Committee of Kyungpook National University (Approval No. 2019-0104-01). C57BL/6J mice purchased from The Jackson Laboratory were used for all *in vivo* experiments. Mice were allowed free access to standard laboratory chow and tap water in a climate-controlled room with a 12 h light/dark cycle. Anesthesia was induced by i.p. injection of 250 mg/kg 2,2,2-tribromoethanol (Sigma–Aldrich, St. Louis, MO, USA) at a dosage of 250 mg/kg in survival procedures and 400 mg/kg in non-survival procedures.

### Minocycline and SB-3CT Treatment

For *in vivo* studies, the minocycline treatment regimen was modified from a previously described study (Scholz et al., 2015). Mice received i.p. injections of minocycline (45 mg/kg; Sigma–Aldrich, M9511), SB-3CT (10 mg/kg; EMD Millipore, Temecula, CA, USA, S1326), a selective MMP-2, 9 inhibitor, or normal saline vehicle with two initial twice-daily injections starting 1 day before CNV induction (Day -1), and one daily injection for 7 days beginning on the day of CNV induction (Day 0).

### Laser-Induced CNV Model

Eight-week-old male C57BL/6J mice were used for experiments. A 532 nm OcuLight GLx Laser System (IRIDEX Corporation, Mountain View, CA, USA) was used to generate lesions. According to previous studies (Yanai et al., 2014; Hasegawa et al., 2017), four lesions were induced for neovascular leakage and CNV size measurements, and 10 lesions were induced for gelatin zymography. The laser was set to the following parameters: 100 mW power, 100 μm spot size and 0.1-s pulse duration. The appearance of a bubble at the site of photocoagulation signified disruption of the Bruch’s membrane. After sacrificing mice, eyes were enucleated and fixed for 30 min. The whole retinas were then separated from the underlying RPE/choroid for whole-mount. For gelatin zymography, unfixed retina and RPE/choroid tissues were snap-frozen in liquid nitrogen for later analysis.

### Choroidal Flat-Mount Preparation

At 7 days after CNV induction (Day +7), eyes were enucleated and fixed in 4% paraformaldehyde for 30 min. Retinas were then removed from the choroid and sclera to generate choroidal flat mounts. Alexa Fluor 488-conjugated to isolectin B4 (1:100; Invitrogen, Carlsbad, CA, USA, I21411) was used to stain the eyecups overnight at 4°C. Flat-mount images were captured using an LSM 800 fluorescence microscope with an Airyscan detector (Carl Zeiss, Oberkochen, Germany). ImageJ software was used to measure CNV lesion size, according to protocols described in previous studies (Yanai et al., 2014; Hasegawa et al., 2017).

### Immunohistochemical Staining

At 6 h and 1 day post-CNV induction, enucleated mouse eyes were fixed in 4% paraformaldehyde for 1 h at room temperature, cryoprotected in 30% sucrose at 4°C overnight, and embedded in OCT compound. Eyes were cryosectioned to 15 μm thickness. For immunofluorescence, sections were blocked in a blocking buffer (0.5% Triton, 0.2% BSA, and 5% donkey serum in PBS) for 1 h at room temperature and subsequently incubated with primary antibodies overnight at 4°C. After washing, samples were incubated with secondary antibodies for 1 h at room temperature. Goat anti-Iba1 antibody (1:250; FUJIFILM Wako Pure Chemical Corporation, Osaka, Japan, 011-27991), rabbit anti-TMEM119 (1:500, Synaptic Systems, Göttingen, Germany, 400 002), rat anti-CD31 (1:100, BD Biosciences, NJ, USA, 553370), and rabbit anti-MMP-9 antibody (1:100; Abcam, Cambridge, MA, USA, ab38898) were used for primary antibodies, and Alexa Fluor 488-conjugated donkey anti-goat antibody (1:500; Thermo Fisher Scientific, Waltham, MA, USA, A11055), Alexa Fluor 594-conjugated donkey anti-rabbit antibody (1:500; Thermo Fisher Scientific, A21207), and Alexa Fluor 647-conjugated donkey anti-rat antibody (1:500; Jackson Immuno Research Laboratories, INC., PA, USA, 712-605-15) were used for secondary antibodies.

### Fluorescein Angiography

A Micron IV Retinal Imaging Microscope (Phoenix Technology Group, Pleasanton, CA, USA) was used to capture fluorescein images. After anesthesia and pupil dilation, images were obtained 3–5 min (early phase) and 7–10 min (late phase) after i.p. injection of 0.1 ml 2% fluorescein sodium (Akorn, Lake Forest, IL, USA). A previously described scheme was used to grade the hyperfluorescent lesions: faint or mottled fluorescence without leakage was scored as 0 (no leakage); hyperfluorescence without any increase in size or intensity between early and late phases was scored as 1 (mild leakage); hyperfluorescence with constant size but increasing intensity was scored as 2A (moderate leakage); and hyperfluorescence with increasing size and intensity was scored as 2B (clinically significant leakage) (Yanai et al., 2014; Hasegawa et al., 2017).

### Gelatin Zymography

Retinal and RPE/choroid tissues were homogenized in lysis buffer (25 mM Tris-HCl buffer, 100 mM NaCl, 1% Nonidet P-40 (NP-40), and Complete Mini EDTA-free Protease Inhibitor Cocktail tablets; Sigma–Aldrich (11836170001) and centrifuged at 14,000 *g* for 10 min. Aliquots of supernatants containing equal amounts of protein (30 μg) were loaded without heating onto 10% SDS-polyacrylamide gels containing 0.1% gelatin (Sigma–Aldrich). Following electrophoresis, gels were washed twice for 30 min in renaturing buffer at room temperature. Gels were then incubated for 48 h in developing buffer at 37°C. After incubation, gels were stained with 0.5% Coomassie blue (Sigma–Aldrich) for 30 min and then destained for 1 h.

### Western Blotting

Samples were lysed using radioimmunoprecipitation assay (RIPA) lysis buffer (Thermo Scientific, Waltham, MA, USA) with protease and phosphatase inhibitor cocktail (Roche Holding AG, Basel, Switzerland) and centrifuged at 4°C and 14,000 *g* for 20 min. The supernatant was collected, and protein concentration was determined using a BCA kit (Thermo Scientific). The same amount of protein for each sample (20 μg) was loaded on a polyacrylamide gel (10%). Proteins were separated by SDS-PAGE and electro-transferred to PVDF membranes (Bio-Rad). Membranes were blocked using 5% skim milk in PBS with 0.25% Tween-20 (PBST), and subsequently incubated at 4°C overnight with anti-MMP9 (1:1,000; Abcam, ab38898) or β-actin (1:5,000; Thermo Scientific, MA5–15739) antibodies. After washing, membranes were bound to HRP-conjugated secondary antibodies [Donkey IgG anti-rabbit (1:2,000; Cell Signaling Technology, Beverly, MA, USA, 7074s) or anti-mouse (1:2,000; Cell Signaling Technology, 7076s)] and incubated at room temperature for 1 h. Blots were developed using enhanced chemiluminescence (ECL) Western blotting detection reagent (Thermo Fisher Scientific) and analyzed using a MicroChemi system (DNR Bio-imaging Systems, Neve Yamin, Israel).

### Surface Marker Staining for Flow Cytometry

For flow cytometric studies, eight retinas were pooled and minced for digestion with 0.05% TrypLE (Thermo Fisher Scientific, 12604021) for 5 min at 37°C. The samples were filtered through a 40 μm cell strainer (SPL) and stained with Zombie Aqua Kit (1:500; Biolegend, San Diego, CA, USA, 423101). The cells were washed and suspended in cold 0.2% BSA (Thermo Fisher Scientific, A1000801) and blocked with Fc Block^™^ (0.01 μg/ml; BD, Franklin Lakes, NJ, USA, 553142) for 5 min at 4°C. Cell surface staining with CD45 V450 (0.3 μg/ml; BD, 560501) and CD11b PerCP-Cy5.5 (0.2 μg/ml; BD, 550993) was performed in the dark for 60 min at 4°C in buffer. Cells were detected using a BD LSR X-20 flow cytometer (BD) and analyzed with FlowJo^™^ V10 (BD). According to previous studies (Greter et al., 2015; Lee et al., 2019), CD11b and CD45 were used to identify the following cells: CD45^low^ resting microglia (CD11b^+^CD45^low^), CD45^intermediate^ activated microglia (CD11b^+^CD45^int^), and CD45^high^ infiltrating leukocytes (CD11b^+^CD45^high^).

### Patient Samples

Institutional Review Board approval from the Kyungpook National University School of Medicine was obtained for all patient samples used in this study (Approval No. KNUH 2012-04-006-020). The study was conducted following the tenets of the Declaration of Helsinki. Informed consent was obtained from all study participants. Aqueous humor samples were collected from consenting patients undergoing cataract surgery in the control group and treatment-naïve patients with neovascular AMD in the disease group.

### Statistical Analysis

Statistical analyses were performed using Prism 6.0 (GraphPad Software, San Diego, CA, USA). Results are expressed as mean ± SEM. Comparison of two groups was performed using an unpaired non-parametric Mann–Whitney U-test. Multiple-group comparison was performed by Tukey’s multiple comparison test. Statistical significance was defined as *P* < 0.05.

## Results

### Elevation of MMP-9 and MMP-2 Activities in Aqueous Humor From Neovascular AMD Patients

**Figure 1:**
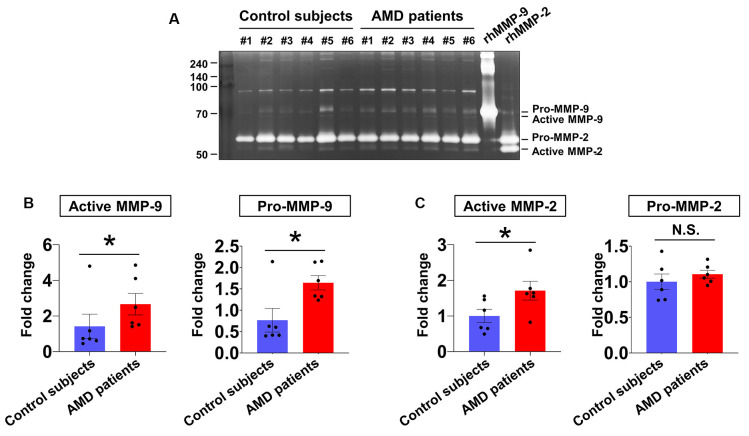
Aqueous humor matrix metalloproteinase (MMP) enzymatic activities in neovascular age-related macular degeneration (AMD) and senile cataract control subjects. **(A)** Gelatin zymography to detect MMP activity (pro-MMP-9, 95 kDa; active MMP-9, 82 kDa; pro-MMP-2, 62 kDa; active MMP-2, 56 kDa). **(B)** Relative abundances of pro-MMP-9 and active MMP-9 were quantified by densitometry. Active MMP-9 was elevated in aqueous humor samples from all six neovascular AMD patients compared with age-matched control subjects. *P < 0.05. **(C)** While active MMP-2 levels were increased in neovascular AMD group relative to control, pro-MMP-2 levels were unchanged. N.S.: not-significant.

In our first series of experiments, the activities of MMP-9 and MMP-2 in aqueous humor samples from six patients with neovascular AMD and six age-matched control subjects were analyzed (Figure 1). Active MMP-9 (Figure 1B, left panel) and active MMP-2 (Figure 1C, left panel) levels were increased in the neovascular AMD group relative to control. Furthermore, pro-MMP-9 (Figure 1B, right panel) was increased in the neovascular AMD group relative to control, but pro-MMP-2 was unchanged (Figure 1C, right panel).

### Early MMP-9 Activity in the CNV Mouse Model

**Figure 2:**
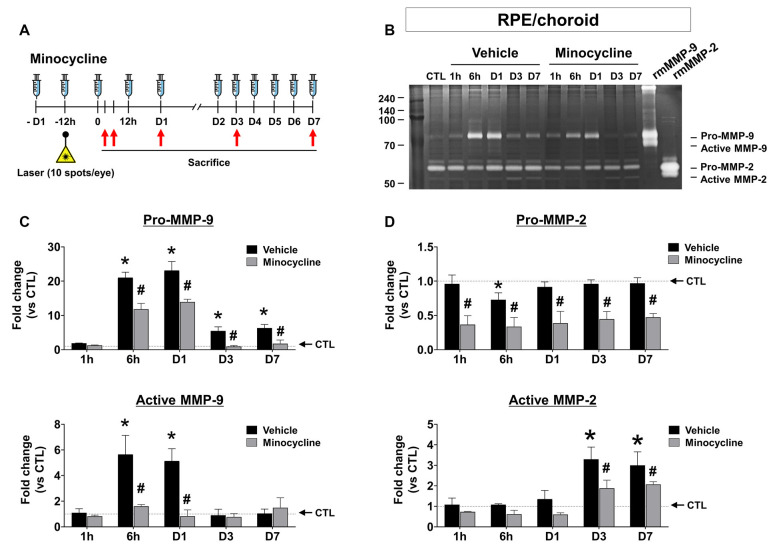
Time-dependent changes of MMP activity and effect of minocycline on MMP activity inretinal pigment epithelium (RPE)/choroid tissues from laser-induced choroidal neovascularization (CNV) mice. **(A)** Experimental design of minocycline injection and induction of CNV with laser photocoagulation. **(B)** Representative image of gelatin zymography to visualize RPE/choroid MMP activity. Lane CTL, non-CNV control; 1 h, 6 h, D1, D3, and D7 after CNV induction. **(C)** Quantification of gelatin zymography of pro-and active MMP-9 in panel **(B)**. Enzymatic activity was measured by band intensity. **(D)** Quantification of gelatin zymography of pro-and active MMP-2 in panel **(B)**. Data are expressed as mean ± SEM. *P < 0.05 vs. CTL; ^#^*P* < 0.05 vs. Vehicle. *n* = 6 eyes/group.

Next, we investigated the spatial-temporal enzymatic activities of MMP-2 and -9 in the CNV mouse model using gel zymography. In the RPE/choroid, the band intensities of pro-MMP-9 and active MMP-9 dramatically increased 6 h after CNV induction, which was maintained through Day 1, followed by a subsequent decrease of active MMP-9 to basal levels by Day 3 post-CNV induction (Figure 2C). Active MMP-2 increased in RPE/choroid tissue from CMV mice at Days 3 and 7, although pro-MMP-2 was not significantly changed (Figure 2D). In retinas from CNV mice, no active MMP-9 or MMP-2 was observed (Supplementary Figure 1). Although MMP-9 Western blots of RPE/choroid tissue exhibited similar time-dependent patterns to gelatin zymography (Supplementary Figure 2), separate pro- and active MMP-9 bands could not be detected, as with gelatin zymography, which could be why prior studies used gelatin zymography instead of Western blotting in this context (Lambert et al., 2002; Manabe et al., 2005; Kim et al., 2018).

Previous studies have demonstrated that MMP-9 amplifies pathological angiogenesis (Pepper, 2001) and inflammation (Nagase and Woessner, 1999), and microglia are a known source of MMPs, contributing to the inflammatory process (Lively and Schlichter, 2013; Rojewska et al., 2014). Furthermore, recruitment of activated microglia appears as early as 1 h after CNV induction (Karlstetter et al., 2015). If microglia contribute to the pathogenesis of CNV by secreting MMP early in the CNV disease process, microglial inhibitors such as minocycline could suppress MMP-9 activities, suppressing the development of CNV. Thus, we confirmed the effect of minocycline over time in the CNV mouse model (Figures 2C,D). In the RPE/choroid, minocycline significantly suppressed both pro and active MMP-9 (Figure 2C), as well as active MMP-2 (Figure 2D, lower panel) at Days 3 and 7.

### Early Microglial Activation and MMP-9 Expression in CNV Mice

**Figure 3:**
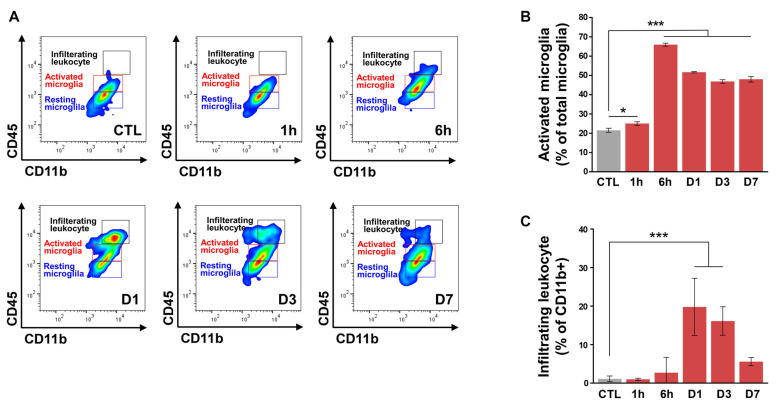
Flow cytometry of retinal cells from control and choroidal neovascularization (CNV) retinal cells to detect microglial activation and infiltrating leukocytes.** (A)** Single live cells were selected and sorted according to their CD11b and CD45 status. Plot CTL, non-CNV control; plot 1 h, 6 h, D1, D3, and D7 after CNV induction. **(B)** The ratio of activated microglia (CD11b^+^CD45^int^) to total microglia started to increase very early after CNV induction at 1 h, peaked at 6 h, and remained significantly increased through D7. **(C)** Compared with the control, the proportion of infiltrating leukocytes (CD11b^+^CD45^high^) in CD11b^+^ cells increased on D1 and D3. *P < 0.05, ***P < 0.001 vs. CTL. *n* = 8 mice/experimental group in three independent experiments.

In addition to microglia, MMPs are also present in leukocytes (Nuttall et al., 2007), although in the laser-induced CNV model, leukocyte infiltration peaks at 2–3 days after CNV induction (Sakurai et al., 2003; Tsutsumi et al., 2003). Thus, we evaluated time-dependent changes in a retinal population of activated microglia and infiltrating leukocytes from CNV mice (Figure 3A). Resident resting microglia (CD11b^+^CD45^low^) were abundant in control mice. The proportion of activated microglia (CD11b^+^CD45^int^) began to increase very early, beginning by 1 h post-CNV induction, peaking at 6 h post-CNV, and maintaining elevation until Day 7 (Figure 3B). The above change was similar to our finding that active MMP-9 increased in the RPE/choroid. However, the proportion of infiltrating leukocytes (CD11b^+^CD45^high^) in CD11b^+^ cells increased at Day 1 and Day 3 and subsequently decreased to basal levels by Day 7 (Figure 3C). This finding is similar to a previous study demonstrating that recruitment of macrophages to CNV lesions occurs 3 days post-CNV induction and subsequently decreases (Jawad et al., 2013).

**Figure 4:**
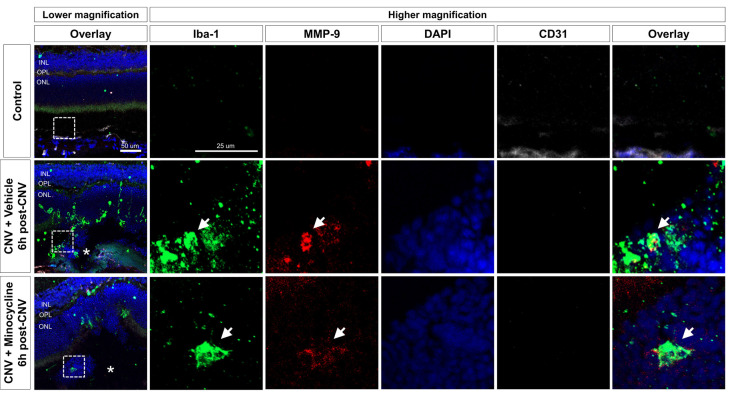
Activated microglia and upregulated MMP-9 expression at 6 h after CNV induction. Immunohistochemistry data of Iba-1^+^ cells (green), MMP-9 (red), and CD31 (white). At 6 h post-CNV induction, Iba-1^+^ amoeboid cells were suggestive of activated microglia aggregation on CNV lesions, indicated by asterisks. Fewer activated microglia were localized to CNV lesions in the minocycline group relative to vehicle control. Furthermore, at higher magnifications, intracellular MMP-9 signal was present in Iba-1^+^ cells, which were CD31^−^. Scale bar: 100 μm. INL, inner nuclear layer; OPL, outer plexiform layer; ONL, outer nuclear layer.

Next, to evaluate the localization of activated microglia with upregulated MMP expression, we conducted immunofluorescence staining of cryosections at 6 h (Figure 4) and 1-day post-CNV induction (Supplementary Figures 3, 4). Activated microglia with amoeboid morphology were aggregated on CNV lesions, and MMP-9 was increased in CNV lesions. Activated microglia in CNV lesions were decreased in the minocycline group relative to the vehicle group. Furthermore, intracellular MMP-9 was present in Iba-1^+^ cells in CNV lesions, which were CD31^−^ (Figure 4). Also, all Iba-1^+^ cells localized to CNV lesions were positive for TMEM119, a specific microglial marker (Bennett et al., 2016; Supplementary Figure 4). Taken together, these findings suggested that microglial activation and MMP-9 expression occurred soon after CNV induction.

### SB-3CT Alleviation of CNV Severity

**Figure 5:**
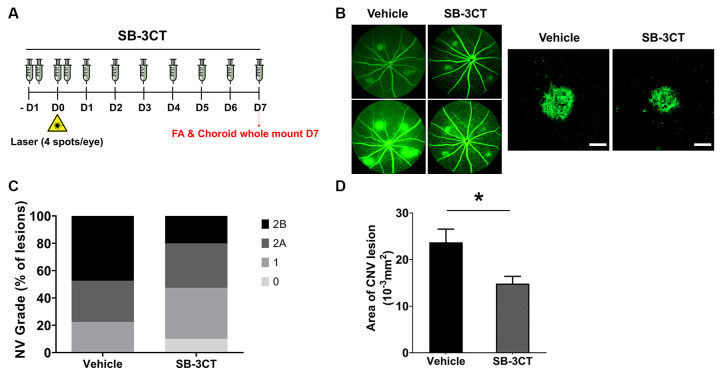
Effect of SB-3CT on neovascular activity in the laser-induced CNV. **(A)** Experimental design of the SB-3CT injection and CNV induction with laser photocoagulation. **(B)** At 7 days after CNV induction, CNV lesion grading was conducted in control and SB-3CT groups. **(C)** The proportion of grade 2B lesions, which exhibit clinically significant leakage, decreased in mice treated with SB-3CT relative to the vehicle. **(D)** CNV lesion size decreased in mice treated with SB-3CT compared with vehicle, as assessed in choroidal flat mounts stained with fluorescent isolectin B4. *P < 0.05. *n* = 48 lesions/group. Scale bar: 100 μm.

To determine if MMP inhibition could alleviate CNV, we evaluated the effects of SB-3CT, a selective MMP-9 and MMP-2 inhibitor (Shin et al., 2016). To evaluate whether early MMP-9 inhibition could affect CNV severity, the SB-3CT administration started 1 day before CNV induction and was maintained until Day 7 (Figure 5A). The proportion of grade 2B lesions, defined as exhibiting clinically significant vascular leakage, decreased in CNV mice treated with SB-3CT relative to vehicle-treated CNV mice (Figures 5B,C). Furthermore, CNV lesion size decreased in mice treated with SB-3CT compared with control mice, as assessed with choroidal flat mounts (Figures 5B–D). This suggested that SB-3CT alleviated neovascular leakage and vascular outgrowth, which could have been modulated by early suppression of MMP-9 from microglia. Similarly, minocycline, a known microglial inhibitor (Yrjanheikki et al., 1999), also decreased neovascular leakage and vascular outgrowth in CNV lesions (Supplementary Figure 5).

These findings suggested that suppression of MMP-9 secreted from microglia could attenuate neovascular lesion formation in CNV. In particular, MMP-9 is involved in the breakdown of the parenchymal basement membranes surrounding blood vessels (Agrawal et al., 2006). This process can damage the Bruch’s membrane and blood-retinal barrier, with accompanying neovascular outgrowth which occurs similarly in the blood-brain barrier (Rosenberg, 2002). Thus, modulation of microglial MMPs could be a targeted approach to CNV treatment.

## Discussion

MMP-9 and MMP-2 are essential to cleavage of collagen type IV, the major component of the basement membrane, and in the context of cancer biology, they are activated at the onset of angiogenesis, resulting in invasive tumor growth (Pittayapruek et al., 2016). However, microglial MMP activity has not previously been evaluated, particularly in the context of CNV.

The normal healthy retina lacks infiltrating leukocytes and has many resident microglia (Liyanage et al., 2016), which is consistent with our flow cytometry data. The proportion of activated microglia increased very early from 1 h post-CNV induction and remained increased until Day 7. By contrast, only a small proportion of infiltrating leukocytes was detected before Day 1. These findings are consistent with a previous study, which demonstrated that CX3CR1-positive microglia migrate to laser-induced lesions as early as 1 h after CNV induction (Karlstetter et al., 2015).

Regarding time-dependent changes of MMP activity in the laser-induced CNV model, it is notable that active MMP-9 increased at very early time points, beginning at 1 h post-CNV induction in the RPE/choroid, which correlated with the increase of microglial activation identified by our FACS data. These results were consistent with a previous study, which demonstrated intense MMP-9 staining in CNV lesions at Day 3, although this study did not evaluate MMP-9 expression at early time points before Day 3 (Lambert et al., 2002).

In the CNV model, active RPE/choroid MMP-2 was increased by CNV in the later stages of the disease. Our *in vivo* experimental data are consistent with the results of other previous studies demonstrating that activated MMP-2 was not present before Day 5 after CNV induction (Lambert et al., 2003). Furthermore, Lively et al. reported differential expression of MMPs from microglia activated by different stimuli (Lively and Schlichter, 2013). MMP-9 increased only in LPS-treated microglia, considered classical (M1) activation. Meanwhile, MMP-2 increased only in IL-4-treated microglia, considered alternative (M2) activation. The above study can explain the *in vivo* data showing that increased MMP-2 activity in the RPE/choroid occurred at Days 3 and 7, likely due to the alternative activation of microglia.

Minocycline, an antibiotic with known microglial inhibitory activity (Yrjanheikki et al., 1999), is neuroprotective in models of light-induced retinal degeneration (Scholz et al., 2015), diabetic retinopathy (Krady et al., 2005), and ischemia-reperfusion injury (Ahmed et al., 2017). In addition to known neuroprotective effects of minocycline, the current study demonstrated that minocycline suppressed pathological neovascularization in laser-induced CNV. Minocycline significantly suppressed microglial MMP-9 secretion in the RPE/choroid, especially at early time points. Furthermore, SB-3CT significantly alleviated disease severity, as demonstrated by decreased vascular leakage and CNV lesion size. Active MMP-9 from activated microglia could potentially provide an advantageous microenvironment for CNV sprouting by degrading the Bruch’s membrane, which is attenuated by minocycline.

There are several limitations of the present study, which should be acknowledged to avoid its overinterpretation. First, our data are not sufficient to demonstrate that microglia are the primary source of MMPs, as Iba-1 also labels infiltrating leukocytes. However, immunofluorescence staining demonstrated that Iba-1^+^ cells were also labeled with TMEM119, a microglia-specific marker, pointing to microglia as an important source of MMPs in this context. Furthermore, taken together, the immunofluorescence and FACS data demonstrate that, at least in the early time points of CNV, microglia could play an important role in angiogenesis by secreting MMPs. Second, because it is not possible to remove RPE/choroid tissues from neovascular AMD patients without damaging vision, we used aqueous samples, which are commonly used to measure biomarkers of neovascular chorioretinal diseases (Hsu et al., 2014). Because active MMP-9 in the aqueous humor could originate from multiple sources, RPE/choroid tissues from human cadaver eyes would be optimal and will be investigated in future studies. Third, because our data pointed to the early recruitment of microglia with the increase of active MMP-9, we focused on the relationship between MMP-9 and microglia, which could be more influential in early CNV than MMP-2. Furthermore, because our data demonstrated that minocycline decreased pro-MMP-9 but not active MMP-9 from Day 3, we focused on early time points in the laser-induced CNV model. In future studies, we will evaluate the roles of alternative microglial activation, especially in later time points of CNV. Finally, as this is an observational study, the mechanism underlying MMP-9-induced CNV will be further investigated in future studies.

A previous pilot clinical trial reported that combined therapy with reduced-fluence photodynamic therapy (PDT), intravitreal ranibizumab (0.3 mg), intravitreal dexamethasone, and oral minocycline for neovascular AMD did not have favorable visual outcomes compared with outcomes of combination treatment with PDT and intravitreal ranibizumab (0.5 mg) reported in other studies (Sivaprasad et al., 2011). However, this pilot study evaluated only a single group and used a reduced dose of ranibizumab (0.3 mg). Thus, it is not clear whether that study clearly evaluated the direct effect of minocycline and should therefore be interpreted cautiously. Thus, our group is in the process of organizing a clinical trial to evaluate the effect of minocycline in neovascular AMD.

Taken together, our data demonstrated nearly immediate recruitment of activated microglia and MMP-9 activation in the RPE/choroid, suggesting that activated microglia that aggregate to CNV lesions could contribute to increased MMP-9 activity. Although we cannot exclude infiltrating leukocytes as a potential source of MMPs, macrophage markers did not increase until Day 3 after CNV induction (Zhou et al., 2017). Furthermore, most MMP RNA levels, including MMP-9, are enriched in microglia compared with peripheral leukocytes (Nuttall et al., 2007). In conclusion, the present study demonstrated a potential role of early microglial MMP-9 contributing to CNV, and that modulation of microglial MMP could be a novel putative therapeutic for CNV.

## Data Availability Statement

The original contributions presented in the study are included in the article/Supplementary Material, further inquiries can be directed to the corresponding author.

## Ethics Statement

The studies involving human participants were reviewed and approved by Institutional Review Board approval from the Kyungpook National University School of Medicine. The patients/participants provided their written informed consent to participate in this study. The animal study was reviewed and approved by Animal Care Committee of Kyungpook National University (2019-0104-01).

## Author Contributions

JK, J-HK, JD, JL, KS, and DP: design and conduct of the study. JK, J-HK, JD, JL, KS, and DP: collection of data. J-HK, JL, RY, KS, and DP analysis and interpretation of data. J-HK, JD, JL, and DP: writing the manuscript. J-HK, I-kL, KS, and DP: critical revision of the manuscript. JK, J-HK, JD, JL, RY, I-kL, KS, and DP: final approval of the manuscript. All authors contributed to the article and approved the submitted version.

## Conflict of Interest

JL was employed by company JD Bioscience Inc. The remaining authors declare that the research was conducted in the absence of any commercial or financial relationships that could be construed as a potential conflict of interest.

## References

[B1] Agrawal S. Anderson P. Durbeej M. van Rooijen N. Ivars F. Opdenakker G.. (2006). Dystroglycan is selectively cleaved at the parenchymal basement membrane at sites of leukocyte extravasation in experimental autoimmune encephalomyelitis. J. Exp. Med. 203, 1007–1019. 10.1084/jem.20051342 16585265 PMC2118280

[B2] Ahmed A. Wang L. L. Abdelmaksoud S. Aboelgheit A. Saeed S. Zhang C. L. (2017). Minocycline modulates microglia polarization in ischemia-reperfusion model of retinal degeneration and induces neuroprotection. Sci. Rep. 7:14065. 10.1038/s41598-017-14450-5 29070819 PMC5656679

[B3] Alcazar O. Cousins S. W. Marin-Castano M. E. (2007). MMP-14 and TIMP-2 overexpression protects against hydroquinone-induced oxidant injury in RPE: implications for extracellular matrix turnover. Invest. Ophthalmol. Vis. Sci. 48, 5662–5670. 10.1167/iovs.07-0392 18055817

[B4] Aslanidis A. Karlstetter M. Scholz R. Fauser S. Neumann H. Fried C.. (2015). Activated microglia/macrophage whey acidic protein (AMWAP) inhibits NFkappaB signaling and induces a neuroprotective phenotype in microglia. J. Neuroinflammation 12:77. 10.1186/s12974-015-0296-6 25928566 PMC4417279

[B5] Bennett M. L. Bennett F. C. Liddelow S. A. Ajami B. Zamanian J. L. Fernhoff N. B.. (2016). New tools for studying microglia in the mouse and human CNS. Proc. Natl. Acad. Sci. U S A. 113, E1738–E1746. 10.1073/pnas.1525528113 26884166 PMC4812770

[B6] Chen M. Xu H. (2015). Parainflammation, chronic inflammation and age-related macular degeneration. J. Leukoc. Biol. 98, 713–725. 10.1189/jlb.3RI0615-239R 26292978 PMC4733662

[B7] Connor K. M. SanGiovanni J. P. Lofqvist C. Aderman C. M. Chen J. Higuchi A.. (2007). Increased dietary intake of omega-3-polyunsaturated fatty acids reduces pathological retinal angiogenesis. Nat. Med. 13, 868–873. 10.1038/nm1591 17589522 PMC4491412

[B8] Ferris F. L. Wilkinson C. P. Bird A. Chakravarthy U. Chew E. Csaky K.. (2013). Clinical classification of age-related macular degeneration. Ophthalmology 120, 844–851. 10.1016/j.ophtha.2012.10.036 23332590 PMC11551519

[B9] Gehrs K. M. Anderson D. H. Johnson L. V. Hageman G. S. (2006). Age-related macular degeneration–emerging pathogenetic and therapeutic concepts. Ann. Med. 38, 450–471. 10.1080/07853890600946724 17101537 PMC4853957

[B10] Greter M. Lelios I. Croxford A. L. (2015). Microglia versus myeloid cell nomenclature during brain inflammation. Front. Immunol. 6:249. 10.3389/fimmu.2015.00249 26074918 PMC4443742

[B11] Hanahan D. Folkman J. (1996). Patterns and emerging mechanisms of the angiogenic switch during tumorigenesis. Cell 86, 353–364. 10.1016/s0092-8674(00)80108-7 8756718

[B12] Hasegawa E. Inafuku S. Mulki L. Okunuki Y. Yanai R. Smith K. E.. (2017). Cytochrome P450 monooxygenase lipid metabolites are significant second messengers in the resolution of choroidal neovascularization. Proc. Natl. Acad. Sci. U S A. 114, E7545–E7553. 10.1073/pnas.1620898114 28827330 PMC5594641

[B13] Hsu M. Y. Yang C. Y. Hsu W. H. Lin K. H. Wang C. Y. Shen Y. C.. (2014). Monitoring the VEGF level in aqueous humor of patients with ophthalmologically relevant diseases via ultrahigh sensitive paper-based ELISA. Biomaterials 35, 3729–3735. 10.1016/j.biomaterials.2014.01.030 24484673

[B14] Huang H. Parlier R. Shen J. K. Lutty G. A. Vinores S. A. (2013). VEGF receptor blockade markedly reduces retinal microglia/macrophage infiltration into laser-induced CNV. PLoS One 8:e71808. 10.1371/journal.pone.0071808 23977149 PMC3748119

[B15] Jawad S. Liu B. Li Z. Katamay R. Campos M. Wei L.. (2013). The role of macrophage class a scavenger receptors in a laser-induced murine choroidal neovascularization model. Invest. Ophthalmol. Vis. Sci. 54, 5959–5970. 10.1167/iovs.12-11380 23927892 PMC3771553

[B16] Karlstetter M. Scholz R. Rutar M. Wong W. T. Provis J. M. Langmann T. (2015). Retinal microglia: just bystander or target for therapy. Prog. Retin. Eye Res. 45, 30–57. 10.1016/j.preteyeres.2014.11.004 25476242

[B17] Kim H. S. Vargas A. Eom Y. S. Li J. Yamamoto K. L. Craft C. M.. (2018). Tissue inhibitor of metalloproteinases 1 enhances rod survival in the rd1 mouse retina. PLoS One 13:e0197322. 10.1371/journal.pone.0197322 29742163 PMC5942829

[B18] Krady J. K. Basu A. Allen C. M. Xu Y. LaNoue K. F. Gardner T. W.. (2005). Minocycline reduces proinflammatory cytokine expression, microglial activation and caspase-3 activation in a rodent model of diabetic retinopathy. Diabetes 54, 1559–1565. 10.2337/diabetes.54.5.1559 15855346

[B19] Lambert V. Munaut C. Jost M. Noel A. Werb Z. Foidart J. M.. (2002). Matrix metalloproteinase-9 contributes to choroidal neovascularization. Am. J. Pathol. 161, 1247–1253. 10.1016/S0002-9440(10)64401-X 12368198 PMC1867305

[B20] Lambert V. Wielockx B. Munaut C. Galopin C. Jost M. Itoh T.. (2003). MMP-2 and MMP-9 synergize in promoting choroidal neovascularization. FASEB J. 17, 2290–2292. 10.1096/fj.03-0113fje 14563686

[B21] Lee S. W. de Rivero Vaccari J. P. Truettner J. S. Dietrich W. D. Keane R. W. (2019). The role of microglial inflammasome activation in pyroptotic cell death following penetrating traumatic brain injury. J. Neuroinflammation 16:27. 10.1186/s12974-019-1423-6 30736791 PMC6367831

[B22] Lively S. Schlichter L. C. (2013). The microglial activation state regulates migration and roles of matrix-dissolving enzymes for invasion. J. Neuroinflammation 10:75. 10.1186/1742-2094-10-75 23786632 PMC3693964

[B23] Liyanage S. E. Gardner P. J. Ribeiro J. Cristante E. Sampson R. D. Luhmann U. F.. (2016). Flow cytometric analysis of inflammatory and resident myeloid populations in mouse ocular inflammatory models. Exp. Eye Res. 151, 160–170. 10.1016/j.exer.2016.08.007 27544307 PMC5053376

[B24] Manabe S. Gu Z. Lipton S. A. (2005). Activation of matrix metalloproteinase-9 *via* neuronal nitric oxide synthase contributes to NMDA-induced retinal ganglion cell death. Invest. Ophthalmol. Vis. Sci. 46, 4747–4753. 10.1167/iovs.05-0128 16303975

[B25] Nagase H. Woessner J. F. Jr. (1999). Matrix metalloproteinases. J. Biol. Chem. 274, 21491–21494. 10.1074/jbc.274.31.21491 10419448

[B26] Nuttall R. K. Silva C. Hader W. Bar-Or A. Patel K. D. Edwards D. R.. (2007). Metalloproteinases are enriched in microglia compared with leukocytes and they regulate cytokine levels in activated microglia. Glia 55, 516–526. 10.1002/glia.20478 17216595

[B27] O’Grady A. Dunne C. O’Kelly P. Murphy G. M. Leader M. Kay E. (2007). Differential expression of matrix metalloproteinase (MMP)-2, MMP-9 and tissue inhibitor of metalloproteinase (TIMP)-1 and TIMP-2 in non-melanoma skin cancer: implications for tumour progression. Histopathology 51, 793–804. 10.1111/j.1365-2559.2007.02885.x 18042068

[B28] Okunuki Y. Mukai R. Nakao T. Tabor S. J. Butovsky O. Dana R.. (2019). Retinal microglia initiate neuroinflammation in ocular autoimmunity. Proc. Natl. Acad. Sci. U S A 116, 9989–9998. 10.1073/pnas.1820387116 31023885 PMC6525481

[B29] Okunuki Y. Mukai R. Pearsall E. A. Klokman G. Husain D. Park D. H.. (2018). Microglia inhibit photoreceptor cell death and regulate immune cell infiltration in response to retinal detachment. Proc. Natl. Acad. Sci. U S A. 115, E6264–E6273. 10.1073/pnas.1719601115 29915052 PMC6142210

[B30] Peng B. Xiao J. Wang K. So K. F. Tipoe G. L. Lin B. (2014). Suppression of microglial activation is neuroprotective in a mouse model of human retinitis pigmentosa. J. Neurosci. 34, 8139–8150. 10.1523/JNEUROSCI.5200-13.2014 24920619 PMC6608244

[B31] Pepper M. S. (2001). Role of the matrix metalloproteinase and plasminogen activator-plasmin systems in angiogenesis. Arterioscler. Thromb. Vasc. Biol. 21, 1104–1117. 10.1161/hq0701.093685 11451738

[B32] Pittayapruek P. Meephansan J. Prapapan O. Komine M. Ohtsuki M. (2016). Role of matrix metalloproteinases in photoaging and photocarcinogenesis. Int. J. Mol. Sci. 17:868. 10.3390/ijms17060868 27271600 PMC4926402

[B33] Rojewska E. Popiolek-Barczyk K. Jurga A. M. Makuch W. Przewlocka B. Mika J. (2014). Involvement of pro- and antinociceptive factors in minocycline analgesia in rat neuropathic pain model. J. Neuroimmunol. 277, 57–66. 10.1016/j.jneuroim.2014.09.020 25304927

[B34] Rosenberg G. A. (2002). Matrix metalloproteinases in neuroinflammation. Glia 39, 279–291. 10.1002/glia.10108 12203394

[B35] Roychoudhury J. Herndon J. M. Yin J. Apte R. S. Ferguson T. A. (2010). Targeting immune privilege to prevent pathogenic neovascularization. Invest. Ophthalmol. Vis. Sci. 51, 3560–3566. 10.1167/iovs.09-3890 20164456 PMC2904009

[B36] Sakurai E. Anand A. Ambati B. K. van Rooijen N. Ambati J. (2003). Macrophage depletion inhibits experimental choroidal neovascularization. Invest. Ophthalmol. Vis. Sci. 44, 3578–3585. 10.1167/iovs.03-0097 12882810

[B37] Scholz R. Sobotka M. Caramoy A. Stempfl T. Moehle C. Langmann T. (2015). Minocycline counter-regulates pro-inflammatory microglia responses in the retina and protects from degeneration. J. Neuroinflammation 12:209. 10.1186/s12974-015-0431-4 26576678 PMC4650866

[B38] Shin J. A. Kim H. S. Vargas A. Yu W. Q. Eom Y. S. Craft C. M.. (2016). Inhibition of matrix metalloproteinase 9 enhances rod survival in the S334ter-line3 retinitis pigmentosa model. PLoS One 11:e0167102. 10.1371/journal.pone.0167102 27893855 PMC5125676

[B39] Sivaprasad S. Patra S. DaCosta J. Adewoyin T. Shona O. Pearce E.. (2011). A pilot study on the combination treatment of reduced-fluence photodynamic therapy, intravitreal ranibizumab, intravitreal dexamethasone and oral minocycline for neovascular age-related macular degeneration. Ophthalmologica 225, 200–206. 10.1159/000322363 21293163

[B40] Tsutsumi C. Sonoda K. H. Egashira K. Qiao H. Hisatomi T. Nakao S.. (2003). The critical role of ocular-infiltrating macrophages in the development of choroidal neovascularization. J. Leukoc. Biol. 74, 25–32. 10.1189/jlb.0902436 12832439

[B41] Wong W. L. Su X. Li X. Cheung C. M. Klein R. Cheng C. Y.. (2014). Global prevalence of age-related macular degeneration and disease burden projection for 2020 and 2040: a systematic review and meta-analysis. The Lancet Global Health 2, e106–e116. 10.1016/S2214-109X(13)70145-1 25104651

[B42] Yanai R. Mulki L. Hasegawa E. Takeuchi K. Sweigard H. Suzuki J.. (2014). Cytochrome P450-generated metabolites derived from omega-3 fatty acids attenuate neovascularization. Proc. Natl. Acad. Sci. U S A 111, 9603–9608. 10.1073/pnas.1401191111 24979774 PMC4084420

[B43] Yrjanheikki J. Tikka T. Keinanen R. Goldsteins G. Chan P. H. Koistinaho J. (1999). A tetracycline derivative, minocycline, reduces inflammation and protects against focal cerebral ischemia with a wide therapeutic window. Proc. Natl. Acad. Sci. U S A 96, 13496–13500. 10.1073/pnas.96.23.13496 10557349 PMC23976

[B44] Zhou Y. Yoshida S. Kubo Y. Yoshimura T. Kobayashi Y. Nakama T.. (2017). Different distributions of M1 and M2 macrophages in a mouse model of laser-induced choroidal neovascularization. Mol. Med. Rep. 15, 3949–3956. 10.3892/mmr.2017.6491 28440413 PMC5436148

