# Supplementary material for: Key Role of Microglial Matrix Metalloproteinases in Choroidal Neovascularization
Source: Front Cell Neurosci. 2021 Feb 26;15:638098. doi: 10.3389/fncel.2021.638098 (PMC7954091; doi:10.3389/fncel.2021.638098)
Supplement: Supplementary file 1 [file Data_Sheet_1.doc]

Supplementary Material

# Key Role of Microglial Matrix Metalloproteinases in Choroidal Neovascularization

Juhee Kim^1§^, Jong-Heon Kim^2§^, Ji Yeon Do^1^, Jung Yi Lee^1,3^, Ryoji Yanai^4^, In-kyu Lee^1,5^, Kyoungho Suk^6^, Dong Ho Park^1, 7^*

*** Correspondence:**Dong Ho Park
DongHo_Park@knu.ac.kr


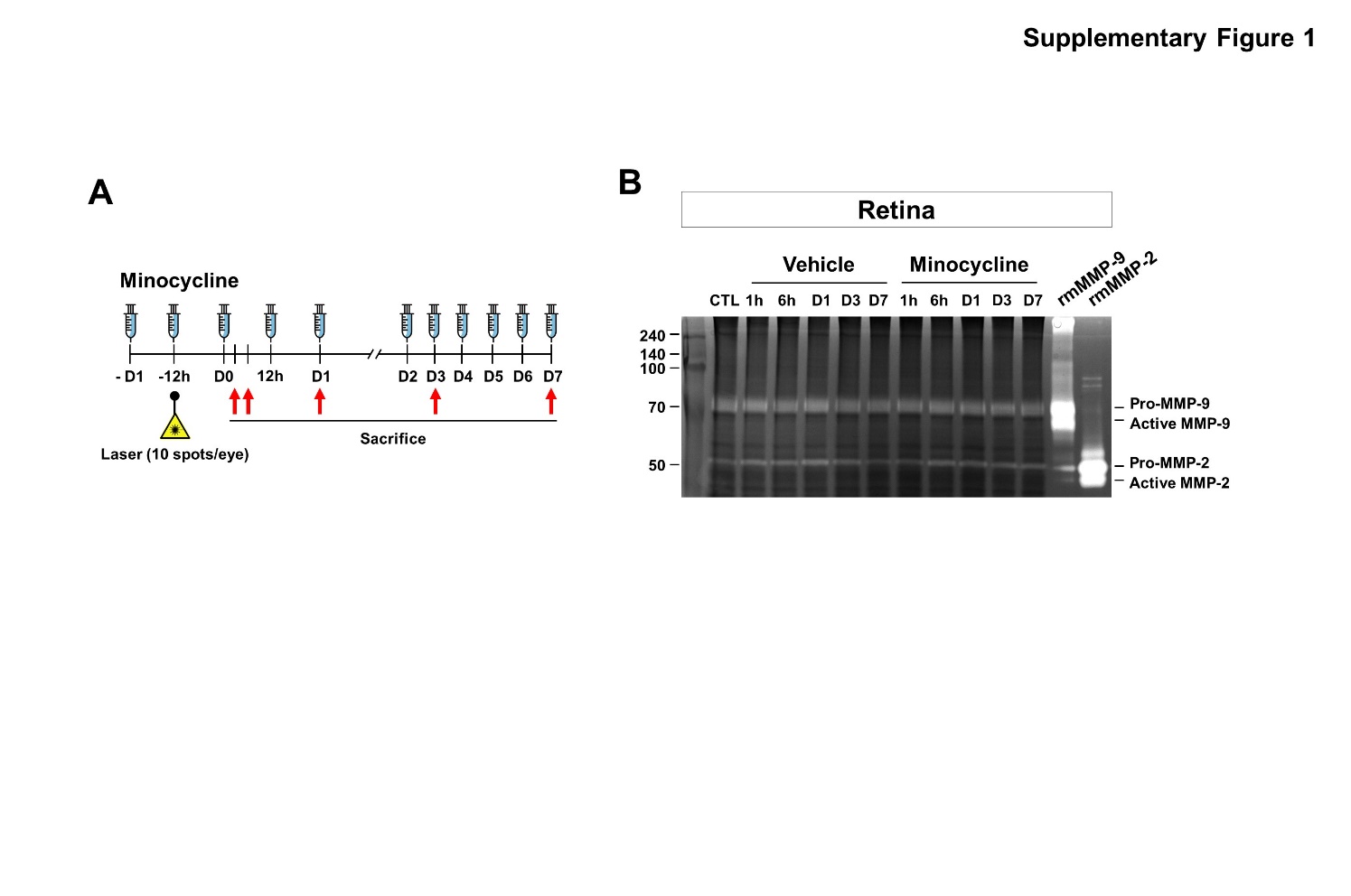


**Supplementary Figure 1.** **(A)** Experimental design of minocycline injection and choroidal neovascularization (CNV) induction with laser photocoagulation. **(B)** Representative image of gelatin zymography used to measure retinal MMP activity. CTL, non-CNV control; 1 h, 6 h, D1, D3, and D7 after CNV induction. n = 6 eyes/group.

**
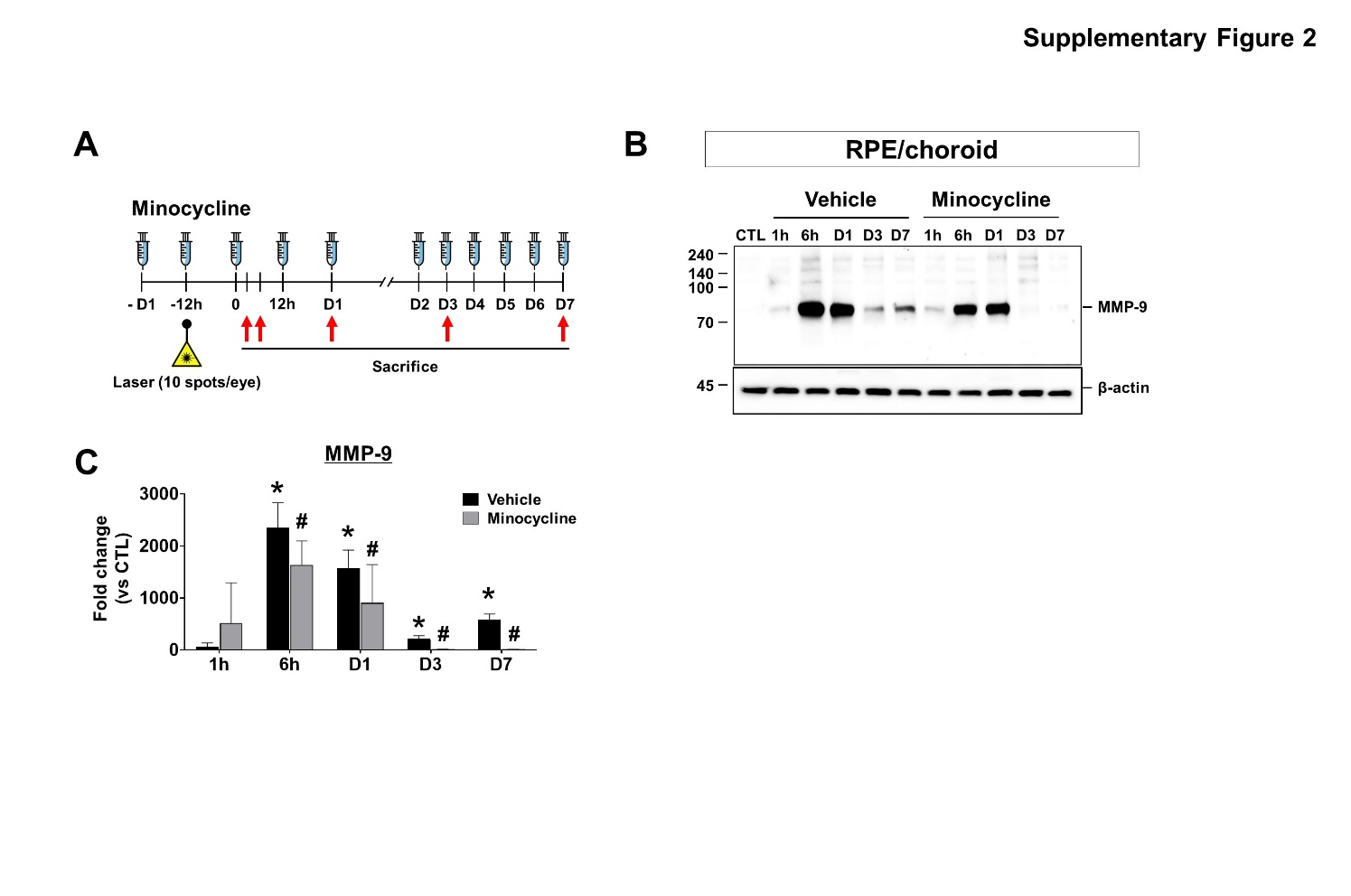
**

**Supplementary Figure 2.** **Time-dependent changes of RPE/choroid MMP-9 protein levels and effect of minocycline on laser-induced choroidal neovascularization (CNV) mice. (A)** Experimental design of minocycline injection and CNV induction with laser photocoagulation. **(B)** Representative images of Western blotting to visualize RPE/choroid MMP-9 protein levels. Lane CTL, non-CNV control; 1 h, 6 h, D1, D3, and D7 after CNV induction. **(C)** Densitometric quantification of MMP-9 protein levels from Western blots. **P* < 0.05 vs. CTL; ^#^*P* < 0.05 vs. Vehicle. n = 6 eyes/group.


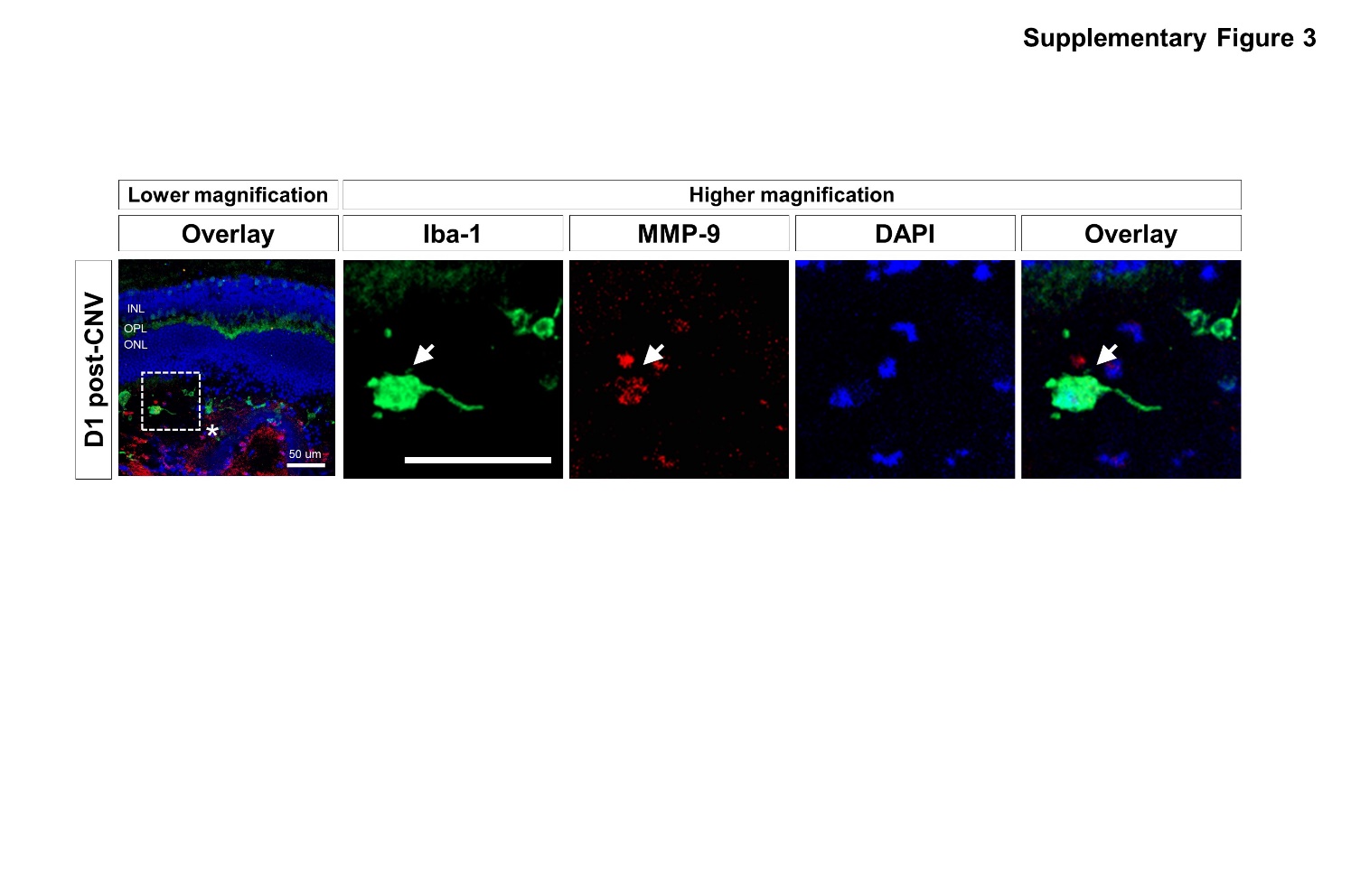


**Supplementary Figure 3.** **Activated microglia and MMP-9 upregulation 1 day after CNV induction.** Immunohistochemistry of Iba-1^+^ cells (green) and MMP-9 (red) revealed microglial MMP-9 expression. At D1 post-CNV induction, Iba-1^+^ amoeboid cells were suggestive of activated microglia aggregation on CNV lesions, indicated by the asterisk. Furthermore, at higher magnifications, intracellular MMP-9 was present in Iba-1^+^ cells. Scale bar: 100 μm. INL, inner nuclear layer; OPL, outer plexiform layer; ONL, outer nuclear layer.


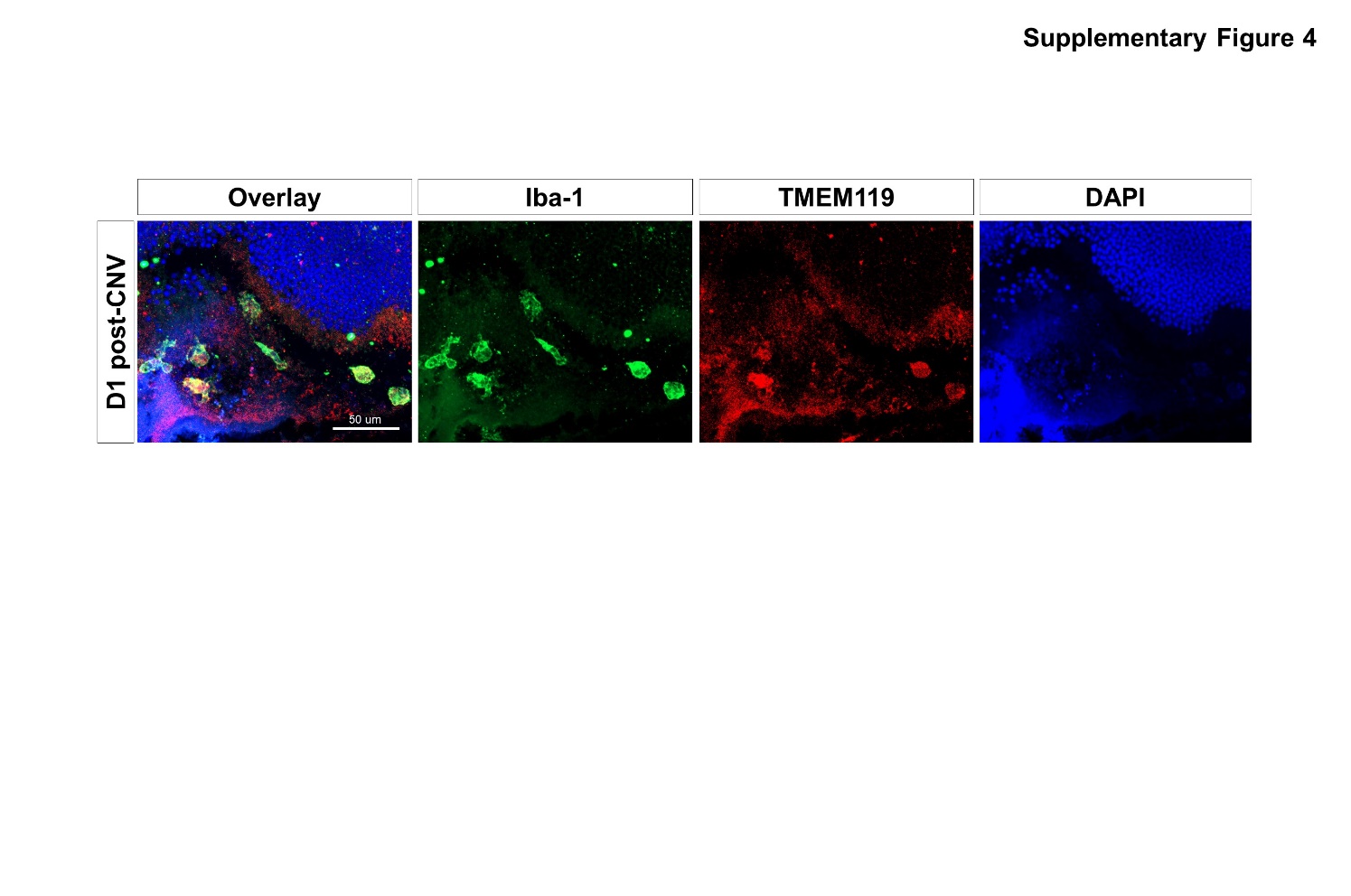


**Supplementary Figure 4.** **Immunohistochemistry of Iba-1^+^ (green) and TMEM119^+^ (red) cells.** All Iba-1^+^ cells localized to CNV lesions were co-labeled with TMEM119, a microglia-specific marker. Scale bar: 50 μm.


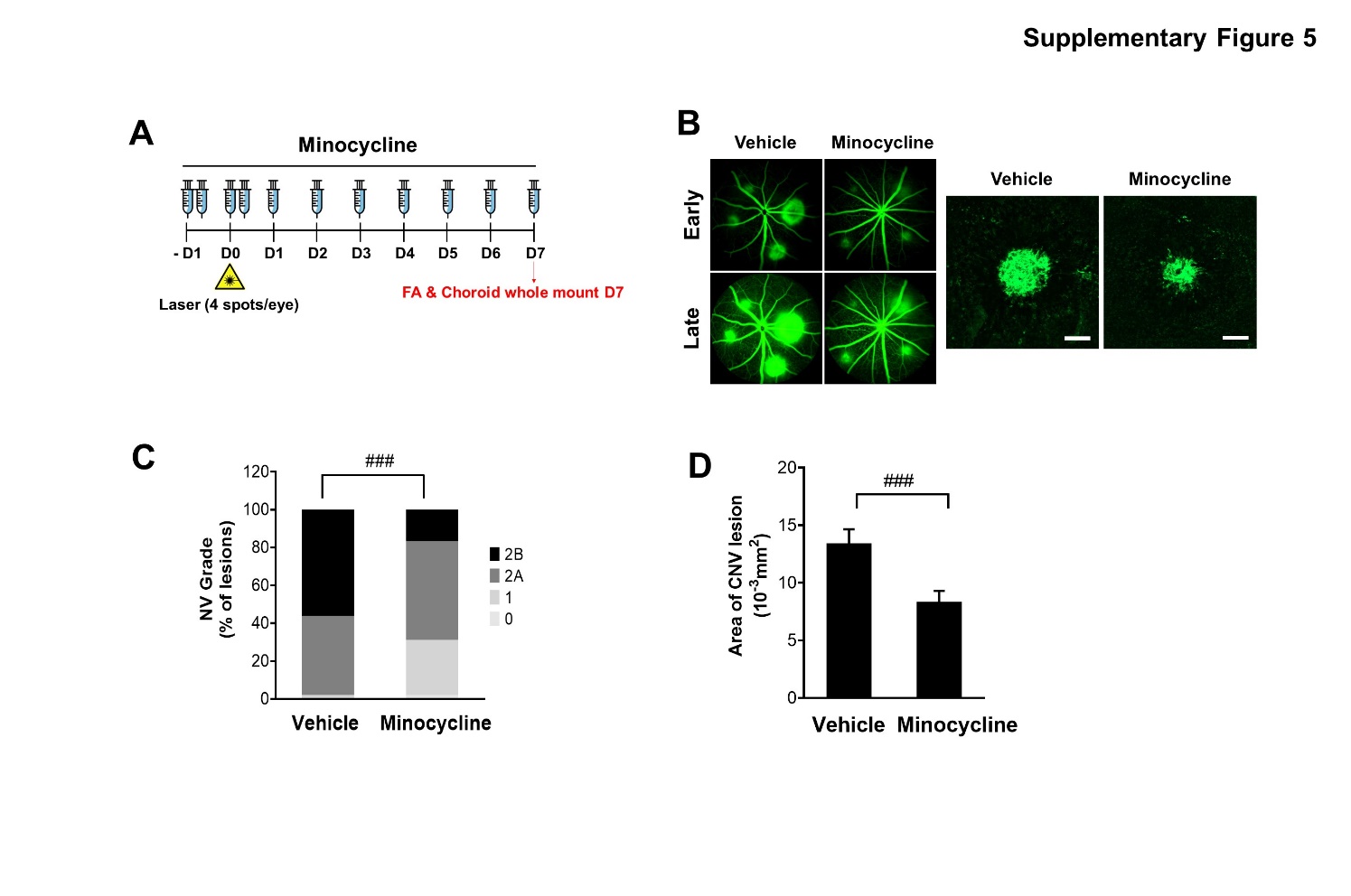


**Supplementary Figure 5.** **Effect of minocycline on laser-induced CNV.** **(A)** Experimental design of minocycline injection and induction of CNV with laser photocoagulation. **(B)** On D7 after CNV induction, CNV lesion grading was conducted in control and minocycline groups. **(C)** The relative proportion of grade 2B CNV lesions, which exhibit clinically significant leakage, decreased in mice treated with minocycline relative to vehicle. **(D)** CNV lesion size on D7 decreased in mice treated with minocycline compared with vehicle, as assessed in choroidal flat mounts stained with fluorescent isolectin B4. ^###^*P* < 0.001. n = 48 lesions/group. Scale bar: 100 μm.
